# Association Between Area-level Risk of Job Instability and Workers’ Health: A Multi-level Analysis Using Population-based Survey Data From Japan

**DOI:** 10.2188/jea.JE20200032

**Published:** 2021-03-05

**Authors:** Takashi Oshio

**Affiliations:** Institute of Economic Research, Hitotsubashi University, Tokyo, Japan

**Keywords:** area-level job instability, multi-level analysis, precarious employment, workers’ health

## Abstract

**Background:**

Precarious job status is negatively related with workers’ health. Research has yet to address whether and to what extent the area-level risk of precarious employment is associated with workers’ health, independently from their job status. We addressed this issue in the present study.

**Methods:**

We estimated multi-level logistic regression models using repeated cross-sectional data comprising 253,048 men and 210,761 women aged 20–59 years who were living in 47 prefectures. This data were obtained from population-based surveys conducted in 2010, 2013, and 2016 in Japan.

**Results:**

For male workers, the estimated odds of reporting poor self-rated health, subjective symptoms, and problems in activities of daily living for those residing in the prefectures in the highest tertile of the proportion of precarious employees were 1.10 (95% confidence interval [CI], 1.01–1.18), 1.12 (95% CI, 1.05–1.19), and 1.15 (95% CI, 1.04–1.28) times, respectively, higher than those living in the prefectures in the lowest tertile, even after controlling for individuals’ job status and key covariates. The results remained largely similar, despite focusing on the sample with information about household income, which was available from the survey, and controlling for it. In contrast, the results indicated that women’s health outcomes were not associated with the prefecture-level proportion of precarious employees.

**Conclusions:**

The area-level risk of precarious employment matters for male workers’ health independently from their job status, underscoring the importance of policy measures aimed to reduce the adverse impacts of precarious employment on workers’ health.

## INTRODUCTION

Workers’ health is closely associated with their job status. Many studies have observed a negative association between precarious employment and workers’ health outcomes, in terms of self-rated health (SRH), psychological distress, and mortality.^1^^–^^6^ Similar and mostly overlapped types of employment, such as temporary, non-permanent, part-time, and more broadly atypical/non-standard employment, have also been noted to be negatively related to workers’ health,^7^^–^^11^ whereas some studies were skeptical about their negative association.^12^^,^^13^

Precarious employment has become a common phenomenon in increasingly “flexible” labor markets in advanced countries over the past decades, reflecting the increased pressure on cost-cutting under global competition and labor market deregulation.^14^^,^^15^ This change in the labor market has important implications for occupational health.^16^^,^^17^ Compared to full-time and permanent workers, precarious employees may be exposed to risks, including job insecurity, low-wage income, and non-coverage of social security programs, which are likely to have adverse impacts on health outcome.^14^^,^^18^^,^^19^ The negative impact of precarious employment has become a notable concern in Japan,^1^^,^^3^^,^^6^ reflecting a steady and substantial increase in the percentage of precarious employees from 15.3% in 1984 to 38.3% in 2019.^20^

Research has yet to address whether and to what extent workers’ health is associated with area-level job instability independently from their job status. Interest in this research topic is inspired by two factors. First, differences have been noted in the associations between workers’ health and actual or contractual security and that of workers’ health and perceived security of employment.^19^ The perceived risk that the current stable job status will be changed to an unstable job status in the near future, or that the current unstable status will continue, is reasonably predicted to adversely impact workers’ health. This perceived risk may be affected by actual employment situations in workplaces, or more broadly, by labor market conditions in areas wherein workers reside and/or work. An observed higher proportion of precarious employees may be perceived to signal a higher risk of becoming or remaining a precarious worker.

Second, many studies have demonstrated that individuals’ health is associated with area-level socioeconomic conditions, despite controlling for their income and other individual socioeconomic status. For instance, area-level income inequality measured using the Gini coefficient has been noted to have a negative association with individuals’ health outcomes.^21^^–^^24^ This association between area-level income inequality and individual health has also been noted in data from Japan.^25^^,^^26^ Income inequality can be interpreted as a signal of income uncertainty, which is considered undesirable by risk-averse individuals, thereby negatively affecting their health. Compared to income inequality, area-level risk of job instability is expected to be more easily and precisely captured, based on the observed proportion of precarious employees in the area where workers live. An observed higher proportion of precarious employees is also likely to be perceived as a signal of higher area-level income inequality or poverty, which in turn may have adverse impact on individuals’ health.

To the best of our knowledge, this study is one of the first attempts to conduct a multi-level analysis for examining the association between area-level job instability and workers’ health. We used pooled cross-sectional data that was obtained through a large-scale, population-based social survey in Japan. We focused on the proportion of precarious employees in the 47 prefectures as a proxy of area-level job instability, and considered three types of health outcomes for workers: SRH, subjective symptoms, and problems in activities of daily living (ADL). We hypothesized that workers’ health is associated with area-level job instability, despite controlling for their job status. We also compared the results between men and women, considering the possibility of gender differences in the association between precarious employment and health, as studies have already observed.^3^^,^^7^^,^^27^

## METHODS

### Study sample

We used a dataset obtained from the Comprehensive Survey of Living Conditions (CSLC), a nationwide population-based survey conducted by the Ministry of Health, Labour and Welfare in Japan. The CSLC, which has been conducted since 1986, is a repeated cross-sectional survey comprising an annual household survey and a triennial health and income survey. The CSLC samples are selected nationwide using a two-stage random sampling procedure. First, approximately 5,400 districts are selected randomly from a total of 940,000 national census districts. Second, approximately 290,000 households are selected randomly from the selected district, based on population size, and the questionnaires about the household and health surveys are mailed to the elected households. The income survey targets a subgroup of the sample of the household and health surveys. In particular, approximately 2,000 residential districts are selected randomly from a total of nearly 5,400 districts that are selected during the first stage of the household and health surveys. The questionnaires of the income survey are then mailed to all households (approximately 30,000 households) who have been selected as the sample of the household and health surveys in each residential district.

In this study, we pooled the data obtained from three waves of CSLC in 2010, 2013, and 2016, when the household, health, and income surveys were conducted. The response rate ranged from 73.7% to 79.6%, and the valid sample rate ranged from 71.8% to 79.4%. We focused on employees (ie, employees excluding managerial and self-employed workers) who were aged 20–59 years old because many firms had set the mandatory retirement age at 60 years. We further constructed two sets of samples: Sample A (comprising the respondents included in the household and health surveys) and Sample B (comprising those included in all three surveys). Sample B, which was a subset of Sample A, additionally contained information about household income. After excluding respondents missing essential information, Sample A comprised 463,809 individuals (253,048 men and 210,761 women), and Sample B comprised 36,994 individuals (19,816 men and 17,178 women). Their sample sizes were 90.2% and 91.5% of the original ones of 514,077 and 40,425 individuals, respectively, before excluding respondents who had missing data.

The CSLC was authorized by the Ministry of Internal Affairs and Communications, which is in charge of all government surveys conducted in Japan, from the statistical, legal, and ethical perspectives, in accordance with the Statistics Law in Japan. We obtained the CSLC data with permission from the Ministry of Health, Labour and Welfare. Therefore, the present study did not require an ethics approval.

### Measures

Regarding job status, the CSLC asked the respondents to report their job status by selecting among the following options: (1) permanent, (2) part-time, (3) short-term and part-time, (4) dispatched, (5) contract, and (6) other. We divided them into “permanent” (1) and “precarious” (2–6), based on Tsurugano et al (2012)^6^ and others, and constructed a binary variable for precarious employment for the regression analysis.

We focused on three types of health outcomes: poor SRH, subjective symptoms, and ADL problems. Regarding SRH, the survey asked the respondents, “What is your current health status? Is it excellent, very good, good, fair, or poor?” We constructed a binary variable for poor SRH referring to the answers “fair” or “poor.” Regarding subjective symptoms, the survey asked, “Have you been feeling ill due to sickness or injury over the past few days?” We constructed a binary variable for subjective symptoms by allocating 1 to the answer “yes”; otherwise, 0 was allocated. Regarding ADL problems, the survey asked, “Do your health problems affect the activities of your daily living?” We constructed a binary variable for ADL problems by allocating 1 to those who answered “yes”; otherwise, 0 was allocated.

As individual-level covariates, we considered age by constructing binary variables for each age group (20–24, 25–29, 30–34, 35–39, 40–44, 45–49, 50–54, and 55–59 years old), educational attainment (junior high school, high school, junior college, college or above, and unanswered), and marital status (married, never married, and divorced/separated). We also considered household income in the regression analysis while using Sample B. To adjust for the household size, we divided the reported household income by the square root of the number of family members. The income data were based on the income and tax records for 1 year prior to each survey year. The data was further evaluated at consumer prices in 2015, based on the Consumer Price Index.^28^

In addition to these individual-level variables, we constructed two prefecture-level variables. There are 47 prefectures, which are key units of the local administration in Japan and have often been used as area units for analyzing regional variations in health, thus reflecting variations in socioeconomic, cultural, and natural features.^29^ The dataset used in this study comprised data from a total of 140 prefectures pooled for 3 years; data from people in one prefecture (Kumamoto) were not collected in 2016 due to the occurrence of the Kumamoto earthquake. The number of respondents in each prefecture ranged from 1,805–7,183, with a mean of 3,313 and a standard deviation of 968. To evaluate job instability at the prefecture level, we calculated the proportion of precarious employees in each prefecture (by dividing the number of precarious employees by the total number of employees in the study sample), divided them into tertiles (low, moderate, and high), and constructed binary variables for each tertile. To capture the overall economic conditions in each prefecture/year, we also considered income per capita in each prefecture/year, based on data regarding the national income^30^ and evaluated at the consumer prices in 2015.

### Analytic strategy

For the descriptive analysis, we first compared health outcomes between permanent and precarious employees for the entire sample, without controlling for other variables. Second, we compared the proportions of each health outcome across the tertiles of the proportion of precarious employees at the prefecture level. After completing these analyses, we estimated four types of multi-level logistic regression models, models 1–4, to explain each health outcome. Models 1 and 2 used Sample A, whereas models 3 and 4 focused on Sample B, which contained information regarding household income. Model 1 considered a binary variable for a respondent’s job status—in particular, precarious employment—as a key regressor, controlling for individual-level covariates (age, educational attainment, and marital status) and prefecture-level income per capita. Model 2 included two binary variables for moderate and high levels of the proportion of precarious employees to model 1. Models 3 and 4, which corresponded to models 1 and 2, respectively, included household income as a regressor. We estimated the models separately for men and women. For all the models, we considered random intercepts at prefecture levels under a multi-level model setup. We used the software package Stata (version 15; Stata Corp, College Station, TX, USA) for the statistical analysis, and considered a *P*-value of 5% or lower to be statistically significant.

Moreover, we conducted three supplementary analyses to evaluate the relevance of the findings obtained from the primary analyses. First, we examined how workers’ health was associated with the interaction between their job status and area-level job instability. None of the association would highlight that area-level job instability is an independent correlate of workers’ health. Second, we examined changes in the results for women if the analysis focused on those without spouses. As emphasized by Kachi et al,^3^ a substantial portion of female precarious employees in Japan seem to be dependent wives, who can financially rely on their husbands. Therefore, we must be cautious while interpreting the relevance of job status for health among women. Third, we divided 47 prefectures into urban and rural areas and compared the estimation results of model 2. The urban area consists of Tokyo, Chukyo, and Kinki areas, which include eight, three, and six prefectures, respectively, while the rural area consists of other 30 prefectures. We considered the possibility that different socio-economic conditions between two areas may affect the observed association between area-level employment instability and health.

## RESULTS

### Descriptive analysis

The key features of the sample used in this study are summarized in Table 1. Precarious employees comprised 30.1% of the total respondents in Sample A. Moreover, the proportion was significantly higher among women (51.7%) compared with that of men (12.1%). In addition to this gender-based difference, Sample B showed patterns that are primarily similar to those in Sample A for other attributes. Compared to women, men had higher educational attainment, and a lower proportion of men were unmarried.

**Table 1.** Key features of respondents

		Sample A			Sample B		
	
		All	Men	Women	All	Men	Women
Employment status, %							
Permanent		69.9	87.9	48.3	68.6	87.4	46.9
Precarious		30.1	12.1	51.7	31.4	12.6	53.1
Married status, %							
Married		62.1	66.3	57.0	61.4	66.6	55.4
Never married		31.2	29.9	32.7	31.5	29.8	33.5
Divorced/separated		6.7	3.7	10.4	7.0	3.5	11.1
Educational attainment, %							
Junior high school		4.0	4.6	3.2	3.8	4.4	3.1
High school		39.3	39.3	39.4	38.6	38.2	39.1
Junior college		21.7	13.5	31.4	22.0	13.2	32.1
College or above		27.0	34.7	17.8	28.9	37.5	19.0
Unanswered		8.1	7.9	8.2	6.7	6.7	6.7
Age, years	*M*	40.9	41.5	40.3	41.7	42.3	40.9
	*SD*	(10.9)	(10.7)	(11.2)	(10.9)	(10.6)	(11.2)
Household income,^a^ ​ million JPY/year	*M*				3.30	3.34	3.25
	*SD*				(1.87)	(1.81)	(1.94)

*N* Year 2010		150,631	81,167	69,464	11,739	6,254	5,485
Year 2013		177,681	96,566	81,115	14,984	7,978	7,006
Year 2014		135,497	75,315	60,182	10,271	5,584	4,687
Total		463,809	253,048	210,761	36,994	19,816	17,178

^a^Household-size adjusted.

Table 2 compares health outcomes based on job status (using Sample A) and household income (using Sample B), without controlling for other variables. Among men, compared to permanent employees, precarious employees faced poorer outcomes, in terms of poor SRH, subjective symptoms, and ADL problems, and had lower household income. The difference based on job status was significantly less apparent among women. The null hypothesis of equality in the proportion of poor SRH could not be rejected at the 5% significance level.

**Table 2.** Health outcomes and household income by employment status^a^

	Permanent	Precarious	Difference	
	
	(A)	(B)	(B–A)	95% CI
Men				
Poor SRH, %	10.1	13.3	3.2	(2.9, 3.6)
Subjective symptoms, %	26.1	30.9	4.9	(4.3, 5.4)
ADL problems, %	7.5	11.3	3.8	(3.4, 4.1)
Household income^b^	3.46	2.50	−0.96	(−1.03, −0.88)
*N*	222,344	30,704		

Women				
Poor SRH, %	9.8	10.0	0.2	(−0.4, 0.1)
Subjective symptom, %	29.1	29.5	0.4	(0.0, 0.8)
ADL problems, %	7.8	8.1	0.3	(0.1, 0.6)
Household income	3.67	2.87	−0.81	(−0.86, −0.75)
*N*	101,750	109,011		

ADL, activities of daily living; CI, confidence interval; SRH, self-rated health.

^a^Data other than household income were included in Sample A, whereas that of household income was included in Sample B.

^b^Annual, million JPY, household-size adjusted.

Table 3 shows how the proportion of each health outcome differed across the prefecture-level tertiles of the proportion of precarious employees. The proportion of precarious employees ranged from 22.8% to 39.0%, with a mean of 30.4% and a standard deviation of 2.9%. Among men, a higher proportionof each health outcome corresponded to a higher tertile of prefecture-level job instability; this was confirmed by the test for trend (*P* < 0.001). In contrast, a positive trend was observed only for subjective symptoms among women. However, it should be noted that these simple prefecture-level comparisons cannot clarify the association between area-level job instability and workers’ health.

**Table 3.** Prefecture-level proportions of precarious employees and health outcomes

Proportion (%) of precarious employees		Men			Women		

Tertile	Range	Poor SRH	Subjective symptoms	ADL problems	Poor SRH	Subjective symptoms	ADL problems
Low	22.8–29.3	10.1	25.6	7.6	10.0	28.4	8.0
Moderate	29.4–31.6	10.4	26.6	8.0	9.9	29.3	8.0
high	31.6–39.0	10.8	27.6	8.3	9.8	30.0	7.8

*P* for trend		<0.001	<0.001	<0.001	0.394	<0.001	0.272

Total	22.8–39.0	10.5	26.7	8.0	9.9	29.3	7.9

ADL, activities of daily living; SRH, self-rated health.

### Regression analysis

Table 4 presents the estimation results of multi-level logistic regression models—models 1 and 2 (using Sample A) and models 3 and 4 (using Sample B)—to explain the probability of poor SRH for men. The upper and lower panels show the results of individual- and prefecture-level variables, respectively. Model 1 indicates that the odds of reporting poor SRH among precarious employees were 1.20 (95% confidence interval [CI], 1.16–1.25) times higher compared to those among permanent employees. This result remained unchanged, despite additionally considering prefecture-level job instability in model 2. The odds of poor SRH in the highest tertile of the proportion of precarious employees were 1.10 (95% CI, 1.01–1.18) times higher than those in the lowest tertile. These results remained largely unchanged in models 3 and 4, which focused on Sample B and allowed us to use information regarding household income. In addition to these key results, we observed that higher household income, having a spouse, and higher educational attainment were positively associated with SRH, whereas prefectural per capita income was uncorrelated with SRH.

**Table 4.** Estimation results of multi-level logistic models to explain poor self-rated health for men^a^

	Sample A (*N* = 253,048)				Sample B (*N* = 19,816)			

	Model 1		Model 2		Model 3		Model 4	

	OR^b^	95% CI	OR^b^	95% CI	OR^b^	95% CI	OR^b^	95% CI
Individual level								
Employment status								
Precarious	1.20	(1.16, 1.25)	1.20	(1.16, 1.25)	1.24	(1.09, 1.41)	1.23	(1.08, 1.41)
Household income					0.94	(0.89, 0.99)	0.94	(0.89, 0.99)
Married status								
Never married	1.59	(1.54, 1.64)	1.59	(1.54, 1.64)	1.52	(1.36, 1.70)	1.53	(1.37, 1.70)
Divorced or separated	1.40	(1.32, 1.49)	1.40	(1.32, 1.49)	1.47	(1.19, 1.83)	1.48	(1.19, 1.84)
Educational attainment								
High school	0.88	(0.83, 0.94)	0.88	(0.83, 0.94)	0.78	(0.64, 0.95)	0.78	(0.64, 0.95)
Junior college	0.90	(0.84, 0.96)	0.90	(0.84, 0.96)	0.84	(0.67, 1.05)	0.84	(0.67, 1.05)
College or above	0.78	(0.74, 0.83)	0.78	(0.74, 0.83)	0.76	(0.62, 0.93)	0.76	(0.62, 0.93)
Unanswered	0.79	(0.74, 0.85)	0.79	(0.74, 0.85)	0.62	(0.48, 0.81)	0.62	(0.48, 0.81)

Prefecture level								
Proportion of precarious employees								
Moderate			1.04	(0.96, 1.12)			1.12	(0.97, 1.30)
High			1.10	(1.01, 1.18)			1.17	(1.02, 1.35)
Income per capita	1.00	(0.97, 1.03)	0.99	(0.96, 1.03)	0.97	(0.92, 1.03)	0.97	(0.92, 1.02)

CI, confidence interval; OR, odds ratio.

^a^Age groups (20–24, 25–29, 30–34, 35–39, 40–44, 45–49, 50–54, and 55–59 years) were controlled for.

^b^Reference categories were “low” (for the proportion of precarious employees) and “permanent” (for employment status). For household income and per-capita income, calculated in response to their one-standard-deviation increases.

Similar results were obtained for subjective symptoms and ADL problems among men, as summarized in Table 5. Similar to poor SRH, the results confirmed that both health outcomes were closely associated with precarious employment. In addition, despite controlling for individual job status, the highest tertile of the proportion of precarious employees raised the odds of poorer health outcome. Model 2 results showed that the odds ratios (ORs) were 1.12 (95% CI, 1.05–1.19) and 1.15 (95% CI, 1.04–1.28), relative to the lowest tertile for subjective symptoms and ADL problems, respectively. At the same time, comparisons between models 1 and 2 revealed that including these variables of prefecture-level job instability did not affect the OR for an individual’s precarious employment status. These results were generally repeated in models 3 and 4.

**Table 5.** Estimation results of multi-level logistic models to explain subjective symptoms and problems in activities of daily living for men

	Sample A (*N* = 253,048)				Sample B (*N* = 19,816)			

	Model 1		Model 2		Model 3		Model 4	

	OR^a^	95% CI	OR	95% CI	OR	95% CI	OR	95% CI
Subjective symptoms								
Employment status								
Precarious	1.19	(1.15, 1.22)	1.18	(1.15, 1.22)	1.09	(0.99, 1.20)	1.09	(0.99, 1.20)
Proportion of precarious employees								
Moderate			1.06	(0.99, 1.13)			1.07	(0.97, 1.19)
High			1.12	(1.05, 1.19)			1.12	(1.01, 1.23)

ADL problems								
Employment status								
Precarious	1.30	(1.25, 1.36)	1.30	(1.25, 1.35)	1.33	(1.16, 1.54)	1.33	(1.15, 1.53)
Proportion of precarious employees								
Moderate			1.08	(0.97, 1.20)			1.14	(0.96, 1.35)
High			1.15	(1.04, 1.28)			1.20	(1.01, 1.42)

ADL, activities of daily living; CI, confidence interval; OR, odds ratio.

^a^Reference categories were “low” (for the proportion of precarious employees) and “permanent” (for employment status).

See Table 4 for other explanatory variables included in regression models.

Compared to the results for men, the association between employment status and health were less apparent for women. Only subjective symptoms were noted to be associated with employment status consistently in all models. No association was observed for poor SRH and the association disappeared while using Sample B for ADL problems. The association with prefecture-level job instability was observed only in model 2 for subjective symptoms.

### Supplementary analysis

The results of the supplementary regression models are presented in eTable 1, eTable 2, and eTable 3. First, we modified models 2 and 4 by adding two interaction terms: (1) precarious employment × a moderate proportion of precarious employees and (2) precarious employment × a high proportion of precarious employees. As seen in eTable 1, none of these interaction terms were associated with any health outcome. In addition, the ORs for precarious employment and tertile variables of the proportion of precarious employees remained mostly unchanged from those in the original models 2 and 4 (see Table 4).

Second, we estimated all models for women by restricting the sample to those without spouses. eTable 2 shows that precarious employment was negatively associated with health in all model specifications, unlike the mixed results in Table 6. However, the association between prefecture-level job instability and health remained unchanged.

**Table 6.** Estimation results of multi-level logistic models to explain health outcomes for women

	Sample A (*N* = 210,761)				Sample B (*N* = 17,178)			

	Model 1		Model 2		Model 3		Model 4	

	OR^a^	95% CI	OR	95% CI	OR	95% CI	OR	95% CI
Poor SRH								
Employment status								
Precarious	0.99	(0.96, 1.02)	0.99	(0.96, 1.02)	0.99	(0.89, 1.10)	1.00	(0.89, 1.11)
Proportion of precarious employees								
Moderate			0.99	(0.95, 1.04)			1.01	(0.88, 1.17)
High			0.98	(0.94, 1.03)			0.94	(0.82, 1.08)

Subjective symptoms								
Employment status								
Precarious	1.05	(1.03, 1.07)	1.05	(1.03, 1.07)	1.08	(1.01, 1.16)	1.08	(1.01, 1.16)
Proportion of precarious employees								
Moderate			1.04	(1.00, 1.09)			0.99	(0.89, 1.10)
High			1.06	(1.01, 1.10)			0.99	(0.89, 1.10)

ADL problems								
Employment status								
Precarious	1.05	(1.01, 1.08)	1.05	(1.01, 1.08)	1.06	(0.94, 1.19)	1.06	(0.95, 1.20)
Proportion of precarious employees								
Moderate			1.01	(0.95, 1.08)			1.01	(0.87, 1.18)
High			0.99	(0.93, 1.06)			0.91	(0.78, 1.06)

ADL, activities of daily living; CI, confidence interval; SRH, self-rated health.

^a^Reference categories were “low” (for the proportion of precarious employees) and “permanent” (for employment status).

See Table 4 for other explanatory variables included in regression models.

Third, we compared the estimation results of model 2 between the urban and rural areas in eTable 3. For men, the association between prefecture-level job instability and health was somewhat more remarkable in the urban area than the rural area. The results in the urban area seem to have dominated the results obtained from the entire sample (see Table 4 and Table 5). For women, the observed association was generally mixed in both of two areas, a result largely similar to what was observed from the entire sample (see Table 6).

## DISCUSSION

We examined how workers’ health outcomes were associated with area-level job instability, an issue that has not been addressed in the related research. Using large-scale, population-based survey data, we focused on the proportion of precarious employees in each prefecture as a proxy of the area-level risk of job instability and examined its correlation with each worker’s health outcomes: poor SRH, subjective symptoms, and ADL problems. The key results and their implications are summarized as follows.

First, we confirmed that precarious employment was negatively associated with workers’ health, a result that has been consistent with general observations in previous studies, despite controlling for household income.^1^^–^^6^ The negative association between precarious employment and health was more notable among men, compared to women. This may be true because a substantial portion of female precarious employees were dependent wives, who can financially depend on their husbands. The supplementary analysis that restricted the female sample to those without spouses revealed a consistently negative relationship between precarious employees and health. Combined, these results confirmed the relevance of employment status for workers’ health.

Second and more importantly, we observed that among men, a higher proportion of precarious employees was negatively associated with health. This result was observed despite controlling for each worker’s employment status; the association between employment status and health remained virtually intact with the inclusion of the variables of prefecture-level proportion of precarious employees. Moreover, the results of the supplementary analysis showed that the interaction between an individual’s employment status and area-level job instability was not associated with workers’ health.

Based on these findings, we can reasonably argue that among men, area-level job instability was associated with workers’ health, even after controlling for their employment status. However, there are two noteworthy factors of this argument. First, the association between area-level job instability and workers’ health was consistently observed only among men. This is probably because precarious employment is increasingly common among women, compared to men, and because this employment status does not necessarily indicate an unfavorable factor for financially-dependent wives. However, this gender difference may decline in the future because the proportion of permanent employees may continue to increase among women. Second, the association between area-level job instability and health was modest. The negative association was observed only among the higher tertile of the proportion of precarious employees, and the OR relative to the lowest tertile ranged between 1.1 and 1.2, not higher than that of precarious employment.

This study has several limitations, in addition to concerns regarding the reliability of self-reported health outcomes. First, the estimated results obtained from our cross-sectional analysis did not reveal details about the causation from area-level job instability to workers’ health, which should be bidirectional in nature.^13^ In addition, there were confounding factors that were not considered in this study. Notably, the prefectures with a similar proportion of precarious employees are likely to have similar demographic, industrial, or geographical characteristics, which determine the prefecture-level structure of job types and hence the demand for precarious employees. If that is the case, a cross-sectional analysis may lead to an overestimated association between precarious employment and health, as already suggested by longitudinal studies that have provided relatively mixed results regarding the association between two variables.^12^^,^^13^

Second, we should be cautious regarding the generalization of the results, which were obtained from survey data in Japan. Cross-country studies have demonstrated that the association between precarious employment and health differ slightly among countries, depending on the labor market and other policies.^5^ This is may be true of the association between area-level proportion of precarious employees and health.

In all, the results are generally consistent with the perspective that a higher proportion of precarious employees is perceived as a sign of a higher risk of job instability, which in turn hurts workers’ health and precarious job status. Policymakers should recognize the harmful health effects of employment precariousness, although more in-depth analysis is needed to identify the mechanism linking area-level risk of job instability to workers’ health.

## APPENDIX A. SUPPLEMENTARY DATA

eTable 1. Estimation results of multi-level logistic models to explain health outcomes for men, including interaction terms

eTable 2. Estimation results of multi-level logistic models to explain health outcomes for women without spouse

eTable 3. Comparing estimation results of Model 2 in Sample A between urban and rural areas
