# Supplementary material for: Association Between Area-level Risk of Job Instability and Workers’ Health: A Multi-level Analysis Using Population-based Survey Data From Japan
Source: J Epidemiol. 2021 Mar 5;31(3):203–9. doi: 10.2188/jea.JE20200032 (PMC7878707; doi:10.2188/jea.JE20200032)
Supplement: Supplementary file 1 [file je-31-203-s001.pdf]

eTable 1. Estimation results of multi-level logistic models to explain health outcomes for men, including interaction terms

		Modified M2		Modified M4	
		OR	95% CI	OR	95% CI
Poor SRH					
Employment status	Precarious	1.19	(1.10, 1.27)	1.03	(0.77, 1.36)
Proportion of precarious employees	Moderate	1.19	(0.96, 1.12)	1.10	(0.94, 1.28)
	High	1.09	(1.01, 1.18)	1.13	(0.97, 1.31)
Interaction terms	Precarious × Moderate	0.99	(0.90, 1.09)	1.21	(0.85, 1.72)
	Precarious × High	1.05	(0.96, 1.15)	1.30	(0.93, 1.81)
Subjective symptoms					
Employment status	Precarious	1.21	(1.15, 1.28)	1.05	(0.86, 1.28)
Proportion of precarious employees	Moderate	1.06	(0.99, 1.13)	1.06	(0.95, 1.18)
	High	1.12	(1.05, 1.20)	1.12	(1.01, 1.24)
Interaction terms	Precarious × Moderate	0.99	(0.92, 1.06)	1.12	(0.87, 1.44)
	Precarious × High	0.95	(0.89, 1.02)	1.00	(0.79, 1.27)
ADL problems					
Employment status	Precarious	1.27	(1.18, 1.37)	1.25	(0.93, 1.69)
Proportion of precarious employees	Moderate	1.07	(0.97, 1.19)	1.15	(0.96, 1.38)
	High	1.15	(1.04, 1.27)	1.16	(0.97, 1.39)
Interaction terms	Precarious × Moderate	1.03	(0.93, 1.14)	0.92	(0.63, 1.35)
	Precarious × High	1.03	(0.94, 1.14)	1.19	(0.84, 1.70)

ADL, activities of daily living; CI, confidence interval; OR, odds ratio; SRH, self-rated health.

eTable 2. Estimation results of multi-level logistic models to explain health outcomes for women without spouse

	Sample A (*N*=90,719)				Sample B (*N*=7,654)			
	Model 1		Model 2		Model 3		Model 4	
	OR	95% CI	OR	95% CI	OR	95% CI	OR	95% CI
Poor SRH								
Employment status								
Precarious	1.14	(1.09, 1.19)	1.14	(1.09, 1.19)	1.18	(1.01, 1.38)	1.19	(1.02, 1.38)
Proportion of precarious employees								
Moderate			1.02	(0.95, 1.09)			1.02	(0.84, 1.24)
High			1.06	(0.99, 1.14)			0.95	(0.79, 1.15)
Subjective symptoms								
Employment status								
Precarious	1.09	(1.06, 1.12)	1.09	(1.05, 1.12)	1.14	(1.03, 1.27)	1.14	(1.03, 1.27)
Proportion of precarious employees								
Moderate			1.04	(0.99, 1.10)			0.97	(0.84, 1.11)
High			1.08	(1.02, 1.14)			0.99	(0.89, 1.10)
ADL problems								
Employment status								
Precarious	1.22	(1.16, 1.28)	1.02	(0.94, 1.11)	1.27	(1.06, 1.51)	1.27	(1.07, 1.51)
Proportion of precarious employees								
Moderate			1.02	(0.94, 1.11)			0.98	(0.77, 1.26)
High			1.06	(0.98, 1.15)			0.94	(0.74, 1.20)

ADL, activities of daily living; CI, confidence interval; OR, odds ratio; SRH, self-rated health.

eTable 3. Comparing estimation results of Model 2 in Sample A between urban and rural areas

	Men (*N*=253,048)				Women (*N*=210,761)			
	Urban (*N*=101,360)		Rural (*N*=151,688)		Urban (*N*=79,530)		Rural (*N*=131,231)	
	OR	95% CI	OR	95% CI	OR	95% CI	OR	95% CI
Poor SRH								
Employment status								
Precarious	1.18	(1.11, 1.25)	1.20	(1.15, 1.26)	1.01	(0.96, 1.06)	0.98	(0.94, 1.02)
Proportion of precarious employees								
Moderate	1.14	(1.01, 1.28)	1.09	(1.01, 1.17)	1.09	(0.95, 1.26)	0.98	(0.92, 1.03)
High	1.33	(1.26, 1.39)	1.07	(0.95, 1.20)	1.11	(0.96, 1.28)	0.93	(0.86, 1.01)
Subjective symptoms								
Employment status								
Precarious	1.18	(1.13, 1.23)	1.18	(1.14, 1.22)	1.04	(1.01, 1.07)	1.06	(1.03, 1.08)
Proportion of precarious employees								
Moderate	1.07	(0.97, 1.18)	1.06	(0.99, 1.14)	0.99	(0.89, 1.10)	1.05	(1.01, 1.10)
High	1.14	(1.09, 1.20)	1.05	(0.95, 1.16)	1.02	(0.92, 1.12)	1.02	(0.95, 1.09)
ADL problems								
Employment status								
Precarious	1.17	(1.02, 1.33)	1.12	(1.01, 1.26)	1.15	(0.91, 1.45)	1.03	(0.95, 1.11)
Proportion of precarious employees								
Moderate	1.43	(1.34, 1.52)	1.08	(0.93, 1.24)	1.27	(1.01, 1.59)	0.90	(0.81, 0.99)
High	1.26	(1.21, 1.32)	1.08	(1.01, 1.15)	1.03	(0.99, 1.07)	1.30	(1.07, 1.59)

ADL, activities of daily living; CI, confidence interval; OR, odds ratio; SRH, self-rated health

Note: Urban=Tokyo (Tokyo, Kanagawa, Saitama, Chiba, Ibaraki, Tochigi, Gunma, Yamanashi), Chukyo (Aichi, Gifu, Mie), and Kinki (Osaka, Kyoto, Hyogo, Shiga, Nara, Wakayama)
